# Fiber Laser-Based Lasso-Shaped Biosensor for High Precision Detection of Cancer Biomarker-CEACAM5 in Serum

**DOI:** 10.3390/bios13070674

**Published:** 2023-06-24

**Authors:** Jie Hu, Enlai Song, Yuhui Liu, Qiaochu Yang, Junhui Sun, Jinna Chen, Yue Meng, Yanwei Jia, Zhiguang Yu, Yang Ran, Liyang Shao, Perry Ping Shum

**Affiliations:** 1 Department of Electrical and Electronic Engineering, Southern University of Science and Technology, Shenzhen 518055, China; 12031313@mail.sustech.edu.cn (J.H.); 12068026@mail.sustech.edu.cn (Y.L.); 12233189@mail.sustech.edu.cn (J.S.); chenjn@sustech.edu.cn (J.C.); shenp@sustech.edu.cn (P.P.S.); 2 Guangdong Provincial Key Laboratory of Optical Fiber Sensing and Communications, Institute of Photonics Technology, Jinan University, Guangzhou 510632, China; lane0527@stu2021.jnu.edu.cn (E.S.); yangqc@stu2020.jnu.edu.cn (Q.Y.); 3 Department of Clinical Laboratory, Guangdong Provincial People’s Hospital, Guangdong Academy of Medical Sciences, Guangzhou 511436, China; 4 State-Key Laboratory of Analog and Mixed-Signal VLSI, Institute of Microelectronics, Faculty of Science and Technology-ECE, Faculty of Health Sciences, MoE Frontiers Science Center for Precision Oncology, University of Macau, Macau 999078, China; yanweijia@um.edu.mo; 5 Medcaptain Medical Technology Co., Ltd., Shenzhen 518055, China; george.yu@medcaptain.com

**Keywords:** optical fiber biosensor, fiber laser, lasso structure, CEACAM5 protein, human serum

## Abstract

Detection of trace tumor markers in blood/serum is essential for the early screening and prognosis of cancer diseases, which requires high sensitivity and specificity of the assays and biosensors. A variety of label-free optical fiber-based biosensors has been developed and yielded great opportunities for Point-of-Care Testing (POCT) of cancer biomarkers. The fiber biosensor, however, suffers from a compromise between the responsivity and stability of the sensing signal, which would deteriorate the sensing performance. In addition, the sophistication of sensor preparation hinders the reproduction and scale-up fabrication. To address these issues, in this study, a straightforward lasso-shaped fiber laser biosensor was proposed for the specific determination of carcinoembryonic antigen (CEA)-related cell adhesion molecules 5 (CEACAM5) protein in serum. Due to the ultra-narrow linewidth of the laser, a very small variation of lasing signal caused by biomolecular bonding can be clearly distinguished via high-resolution spectral analysis. The limit of detection (LOD) of the proposed biosensor could reach 9.6 ng/mL according to the buffer test. The sensing capability was further validated by a human serum-based cancer diagnosis trial, enabling great potential for clinical use. The high reproduction of fabrication allowed the mass production of the sensor and extended its utility to a broader biosensing field.

## 1. Introduction

Cancer is one of the greatest threats to human health [1]. Early screening and accurate diagnosis of cancer are crucial for improving therapeutic effectiveness, preventing tumor recurrence, and thus increasing the survival rate of patients [2]. In the early stages of cancer, a small quantity of biomarkers would release from the tumor into the blood, which indicates the presence and classification of the tumor, for example, the alpha fetal protein (AFP), prostate-specific antigen (PSA), and carcinoembryonic antigen (CEA)-related cell adhesion molecules 5 (CEACAM5). Liquid biopsy is expected to detect these biomarkers from blood/serum and has been clinically approved in the field of dynamic monitoring of tumors and treatment response [3].

In contrast with the routine labeling immunoassay methods, for instance, the enzyme-linked immunosorbent assay (ELISA) and fluorescence immunoassay, label-free biosensors provide real-time affinity reaction for biomarker analysis and eliminate the need for complex sample labeling [4,5,6]. Optical fiber biosensors, due to their high responsivity, compact size, and immunity to electromagnetic interference, have shown good potential in probing cancer biomarkers label-free and in operando [7,8]. For example, a harmonic microfiber Bragg grating was designed to detect cardiac biomarkers (cTn-I) and eliminate the interference of temperature variation on detection results at the same time [9]. A long-period grating (LPG)-based biosensor was designed for label-free, bacteriophage-based detection of *Escherichia coli* (*E. coli*) [10]. An optical fiber nano-optrode based on LPG in reflection configuration was used to detect the human Thyroglobulin (TG), a protein marker of differentiated thyroid cancer [11]. An automated optical assay based on reflection-type LPGs was proposed for the fast and label-free detection of class C β-lactamases [12]. However, the concentration of cancer biomarkers in blood is extremely low at the early stages of cancer, which necessitates the high sensitivity and specificity of the fiber-optic biosensors [13,14].

Various research works have focused on enhancing the sensing capability of fiber biosensors from different perspectives. A variety of sophisticated configurations have been proposed, including microcavities, and tapered fiber (turning point of dispersion), to achieve ultra-high sensitivity [15,16,17,18,19]. Furthermore, micro-(nano-) materials or plasmonic structures on fiber were exploited to realize the local effects of molecular enrichment and electromagnetic field enhancement on the surface of the sensor [20,21,22,23,24]. With the development of various fabrication technologies of patterned micro/nanostructures on fiber end faces, such as self-assembled periodic patterns by microsphere arrays [25,26,27], nanoimprinting, and nano-transfer technology [28,29,30], the research on fiber tip biosensors has gradually become popular. Plasmonic gold nanoparticles (AuNPs) were printed directly onto the end face of the multimode fiber, displaying various patterns of flowers and badges, and can be used to detect SARS-CoV-2 mimetic DNA sequence [31]. Nanosphere lithography was used to fabricate a surface-enhanced Raman scattering (SERS) substrate on an optical fiber tip and sandwiched with functionalized gold nanoparticles that provide additional Raman signal amplification to detect human thyroglobulin [32]. Alternatively, frequency domain demodulation methods based on beat frequency, as well as microwave photonic interrogation, were used for enabling high-resolution sensing [33,34,35,36]. To orchestrate the enhancement in both sensitivity and spectral resolution, various sensing structures with fiber laser demodulation methods also aroused extensive attention [37,38,39,40,41]. Compared with the interference spectrum, the laser has a higher signal-to-noise ratio (SNR) and much narrower linewidth, which is easier for spectral analysis. With a variety of sophisticated and complex designs, the sensitivity of the sensor has been greatly improved. However, the sophistication of sensor preparation hinders the reproduction and scale-up fabrication.

In this paper, a lasso-shaped fiber sensor was proposed and demodulated with fiber ring laser technology for the specific detection of CEACAM5 protein in serum and demonstrated good reproducibility, high detection precision, as well as high specificity. Due to the ultra-high spectral resolution and ultra-narrow linewidth of the laser signal, the sensing capability of the proposed laser sensor can be significantly improved. Functionalization of the probe was realized to detect CEACAM5 with the limit of detection (LOD) of 9.594 ng/mL for the buffer solutions. Human serum samples from a hospital were also tested, and the results demonstrated the good sensing capability of our biosensor, which is comparable to the commercial analytical instrument in clinic. It has great potential as a kind of biomedical in situ monitoring device that can be mass-produced.

## 2. Materials and Methods

### 2.1. Reagents and Apparatus

The optical fiber was the single-mode silica fiber (SMF-28) purchased from Yangtze Optical Fiber Ltd. Co. (Wuhan, China). Here, 98% sulfuric acid, 30% hydrogen peroxide, pure ethanol, phosphate-buffered saline (PBS, pH 7.2–7.4), 25% glutaraldehyde water solution, bull serum albumin (BSA), and placental growth factor (PLGF) were obtained from Sangon Biotech (Shanghai, China). BSA-blocking liquid was purchased from Boster Biological Technology (Wuhan, China), and 99% 3-aminopropyltriethoxysilane (APTES) was purchased from Sigma-Aldrich (Darmstadt, Germany). CEACAM5 protein and anti-CEACAM5 antibody were purchased from Sino Biological (Beijing, China). Deionized water (DI Water) with a resistance of 18.25 MΩ⋅cm used in all experiments was obtained from ultra-pure water equipment. The human serum samples were provided by Guangdong Provincial People’s Hospital (Guangzhou, China). Clinical processes were approved by the Ethics Committees of Guangdong Provincial People’s Hospital (KY-Q-2022-388-01).

### 2.2. Antibody Immobilization

To grant the lasso-shaped fiber sensor with the specificity of aiming at the CEACAM5, anti-CEACAM5 antibodies need to be immobilized on the surface of the fiber. The functionalization of the lasso structure probe is described below. Firstly, the probe was inserted into the piranha solution for 2 h for fiber surface modification and creation of a hydroxyl group. The piranha solution was prepared by hybridizing the 98% sulfuric acid and 30% hydrogen peroxide with a volume ratio of 3:1. After rinsing the sensor with deionized water for 5 min to remove the residual solution, the sensor was washed with ethanol solution 3 times to prevent hydrolysis of silanizing solvent. Subsequently, the fiber probe was immersed in a 5% APTES ethanol solution for 90 min to generate an amino group on the surface. Then the probe was reacted in 5% glutaraldehyde PBS solution for 45 min and aldehyde groups were attached to the surface of the fiber. Next, the probe was soaked in the anti-CEACAM5 antibody solution (50 μg/mL) for 60 min. Thereafter, the BSA blocking solution was used to immerse the probe for 30 min to occupy and block the unbind aldehyde groups on the surface. For validating the functionalization of the fiber, atomic force microscopy (AFM Veeco, Nanoscope-V) was employed to observe the surface morphology of the optical fiber. Soak the fiber in DI water for 1 min and then gently rinse the fiber with DI water for 10 s to ensure that no salt crystals and unbonded biomolecules remain on the fiber surface. After drying for 12 h, the functionalized fiber sensor was imaged with AFM and compared with the optical fiber without biological modification. As a control, non-functionalized fibers were also tested for AFM. After removing the coating layer, the fiber was cleaned by soaking in alcohol and dried for 12 h before being tested for AFM. The instrument model of AFM was “Multimode Nanoscope-V” produced by: Veeco Instruments, USA. The image mode was “scanAsyst”.

### 2.3. Sensor Preparation and Interrogation Setup

**Figure 1:**
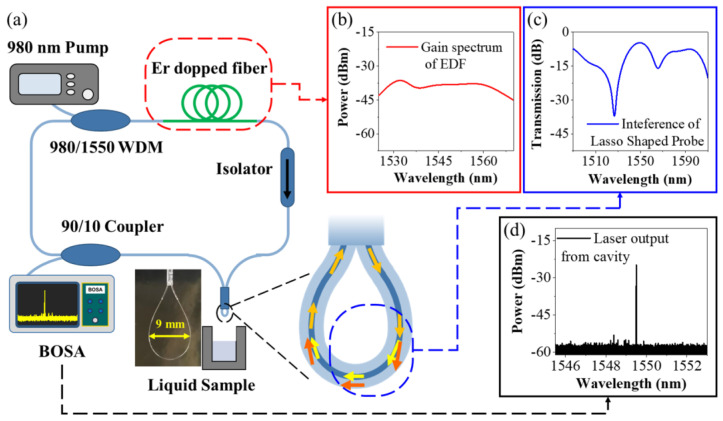
Illustrations of the sensing element and the sensing system of the fiber laser-based lasso-shaped sensor. (**a**) Interrogation setup of fiber laser-based lasso-shaped biosensor; (**b**) Optical signal around 1550 nm generated by erbium-doped fiber under the excitation of 980 nm light; (**c**) The transmission spectrum of a wideband light source of 1490~1610 nm passing through the lasso structure; (**d**) The laser signal with narrow linewidth output from the laser cavity.

Figure 1 shows the sensing element and the sensing system of the fiber laser-based lasso-shaped sensor. A fiber ring laser cavity was used as the experimental setup to interrogate the sensing signal, as shown in Figure 1a. The 980 nm pump laser was launched into the laser cavity through a 980/1550 wavelength division multiplexer (WDM). A section of erbium-doped fiber (EDF, ER80-8/125, Thorlabs, Shanghai, China) with a length of around 40 cm was used as the gain medium. The doped erbium particles in EDF can absorb the incident light at 980 nm and release the light at around 1550 nm as the initial signal of the system. The gain spectrum of EDF under 980 nm excitation light (200 mW) is shown in Figure 1b. The isolator ensured the one-way circular transmission of light in the fiber ring cavity. The optical fiber lasso structure was the filter in the laser cavity, which was also the sensing unit of the system. Figure 1c presents the transmission spectrum of a wideband light of 1490~1610 nm passing through the lasso structure. Because of the interference phenomenon inside the lasso structure (see the detailed analysis below), it has different transmission losses for lights with different wavelengths, with the loss near 1530 nm, in particular, exceeding 30 dB. A 90/10 fiber coupler was used to separate 10% of the light energy out of the cavity (laser output), while the other 90% remained inside the cavity for cycle amplification. The optical signal generated by EDF (1530~1570 nm) was constantly circulating in the cavity, and some wavelengths of light were constantly suppressed due to higher transmission losses. Finally, the remaining laser signal with a narrower linewidth was output from the laser cavity, as shown in Figure 1d, and was launched into an ultrahigh-resolution optical spectrum analyzer (BOSA-light, Argon Photonics Ltd., Zaragoza, Spain, optical resolution of 0.08 pm) for spectrum analyzation.

Our lasso-shaped fiber sensor was derived from the originally existing SMF in the ring cavity, and no more complex operations were added. Firstly, the protective coating of SMF was stripped off, with a range of around 3 cm. Then, this piece of SMF was bent into a lasso-like structure. A silica capillary with an internal diameter of 0.9 mm was used to hold the lasso shape. By pulling the two ends of the fiber, the bending diameter of the lasso structure can be regulated as well as its interference spectra. Here, the bending diameter of 9 mm was chosen (see the inset in Figure 1) and presents a significant interference phenomenon inside the fiber (transmission spectrum in Figure 1 with blue line). UV glue was used to further reinforce the stability of the structure. Compared with previous delicate and fragile microstructures [42,43,44], our sensing device unit is convenient to fabricate and can be repeated to achieve similar sensing performance easily (see sensor characterization below). Lasso-shaped fiber sensors can be inserted into the liquid for in situ detection.

## 3. Results and Discussion

### 3.1. Sensing Characteristics of the Sensing Probe

**Figure 2:**
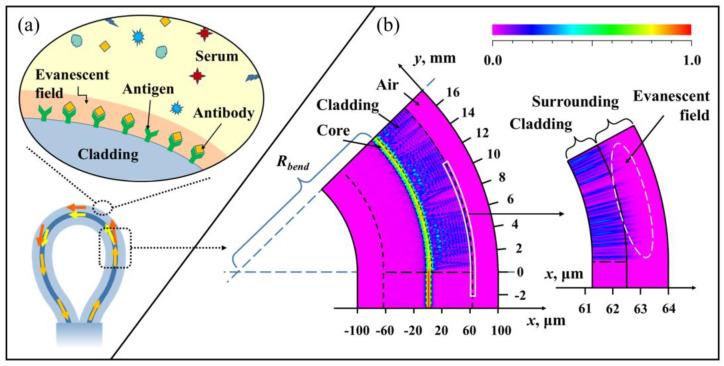
The sensing principle of the lasso-shaped sensor: (**a**) RI perturbation caused by specific binding of proteins on the bent fiber surface are perceived by the fiber cladding mode (or evanescent field). (**b**) The simulated optical field distribution in the straight SMF and bent SMF.

The schematic overview of the lasso-shaped fiber sensor is shown in Figure 2a. When the bending radius (*R_bend_*) is small enough, the light in the fiber core cannot be bound in the core. Part of the optical field energy will leak out of the fiber core into the cladding and propagate forward as cladding modes The cladding modes would interfere with the core mode, forming multimode interference (MMI) [45,46,47].

Numerical simulations have been performed using a commercial software package Rsoft based on the beam propagation method (BPM) for the mode field distribution inside the bent SMF, as shown in Figure 2b. The incident light intensity profile was assumed to be Gaussian. The free space wavelength was set to 1.55 μm. The incident light first enters a straight SMF with a length of 3 mm and then enters a bent SMF (simulation length of 18 mm) with a bend radius of 4.5 mm. The diameter and RI of the SMF core and cladding were 8.2/125 μm and 1.457/1.4447, respectively. The simulation mesh size in the radial direction *X* and propagation direction *Y* were set to be 0.02 μm, and 0.2 μm, respectively. The simulation window size was 160 μm × 21,000 μm. The boundary condition corresponded to a perfectly matched layer condition.

It can be seen from the simulation results that the energy in a straight SMF (*y* = −3~0 mm) is almost bound in the fiber core and stably transmitted forward, while there is almost no energy in the cladding. When the fiber is bent (*y* = 0~16 mm), although the light energy in the fiber core is still the majority, a large amount of light energy is leaked to the cladding as the cladding mode. In the forward transmission process, these cladding modes will also be coupled with the core mode, and the intensity of the light field in the core and cladding region will change every certain distance. For example, in the simulation results, the energy in the fiber core increases again (color identification changes from yellow to red) at the positions of *y* = 8 mm and *y* = 16 mm, while the energy of cladding mode decreases (color identification changes from blue to purple). These phenomena indicate that the coupling between the core mode and the cladding mode occurs during the optical field transmission.

Their different effective refractive indexes (RIs) and paths caused the multi-mode interference at the output of the device. The transmission spectrum (spectrum in Figure 1 with a blue curve) of the lasso structure measured by the experiment also shows a strong interference phenomenon, and the output energy shows obvious energy intensity change with an extinction ratio larger than 10 dB. The resulting phase difference between the cladding mode (m-order) and core mode can be expressed as:

(1) $$∆\varphi_{m}=2\pi \times \frac{{∆n}_{eff}\times L_{eff}}{\lambda }$$

where *L_eff_* was the effective transmission length, $∆n_{eff}$ was the effective RI difference between the core mode and cladding mode, respectively, *λ* was the wavelength. When the phase difference becomes an odd (even) multiple of π, destructive interference (constructive interference) occurs:

(2) $$2\pi \times {∆n}_{eff}\times L_{eff}/\lambda_{dip}=(2k+1)\pi$$

(3) $$2\pi \times {∆n}_{eff}\times L_{eff}/\lambda_{peak}=2k\pi$$

where *k* is a positive integer. Then the resonant wavelength *λ* can be depicted as:

(4) $$\lambda_{dip}=2L_{eff}\times ∆n_{eff}/\left(2k+1\right)$$

(5) $$\lambda_{peak}=L_{eff}\times ∆n_{eff}/k$$

By enlarging the radial *x* = 61~64 μm region of the simulation results, it can be found that the evanescent field (about 1~2 μm in depth) is formed on the near-surface outside the cladding. Therefore, the cladding mode is responsive to near-surface environmental changes. For a lasso-shaped fiber sensor with a fixed bending radius, when the external RI varies, the effective RI of the cladding mode also changes. Thus, the effective RI difference $∆n_{eff}$ will change, which leads to a spectral shift.

**Figure 3:**
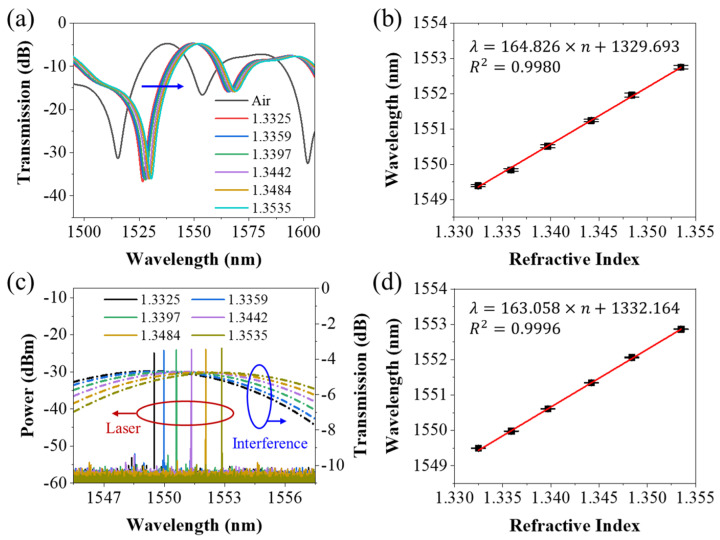
(**a**) Interference spectrum of lasso-shaped fiber sensor when in air and in liquid with RIs varying from 1.3325 to 1.3535; (**b**) The linear fitting of the interference peak wavelength corresponding to RIs; (**c**) Laser spectrum from fiber laser ring cavity with lasso structure in liquid with RIs varying from 1.3325 to 1.3535, and its corresponding interference spectrum; (**d**) The linear fitting of the laser wavelength versus the different RIs.

Using the amplified spontaneous emission (ASE) and optical spectrum analyzer (OSA, Yokogawa, AQ6370D, Shanghai, China) to find the interference spectrum of the lasso structure fiber sensor. Figure 3a shows the transmission spectrum of the lasso structure and its local magnification of the interference peak, when in air and being immersed in solution with RIs varying from 1.3325 to 1.3535. As the surrounding RI increases, a redshift phenomenon can be observed. The linear fitting of the interference peak wavelengths corresponding to RIs was given in Figure 3b. The result showed that the RI sensitivity of interference peak wavelength was 164.826 nm/RIU with good linearity of around 0.9980.

Then the lasso structure was connected to the fiber ring laser cavity as a filter. The lasso structure acts as a filter in the laser cavity. Different transmission losses were introduced for light with different wavelengths in the cavity. Interference dips (peaks) mean a larger (smaller) loss in the laser cavity. Due to energy competition in the laser cavity, the wavelength with the lowest transmission loss in the cavity will eventually output the laser signal. Thus, the wavelength of the laser would be affected by the interference spectrum, i.e.,:

(6) $$\lambda_{Laser}=\lambda_{peak,max}$$

Therefore, sensing based on interference spectrum becomes sensing based on laser wavelength. Figure 3c presents the laser spectrum when the lasso structure was immersed into the liquid with different RIs varying from 1.3325 to 1.3535. As the surrounding RI increases, a noticeable redshift can be observed. The wideband interference spectra under the same conditions are also included in the diagram for comparison with laser signals. The linear fitting of the laser wavelengths versus the different RIs was given in Figure 3d. The results showed a good linearity of around 0.9996. The RI sensitivity of the laser was 163.058 nm/RIU, which was almost the same as that of the interference spectrum. The slight numerical difference can be understood as an error in the experimental process.

Although the sensitivity of both is the same, the interference peaks of the interference spectra under different RIs overlapped with each other, while the offset of the laser spectrum is more distinguishable due to the smaller linewidth of the laser, which can be seen in Figure 3c easily. Moreover, compared with the laser signal, the interference peak of the interference spectrum is much flatter, and the line width is larger. When the highest point of the interference spectrum is selected as the sensing reference point, there will be a large fluctuation. This is also proven by the experimental results, the error bar of each data point in Figure 3b will be larger than that in Figure 3d.

The figure of merit (FOM) can be used to describe this performance of the sensor and was described as:

(7) $$FOM=\frac{S}{∆\lambda_{3dB}}$$

Here, *S* means the sensitivity of the sensor, Δ*λ_3dB_* means the full width at half maximum (FWHM) of the spectrum. According to Equation (7), the FOM parameter of this interference spectrum was 9.76, while that of the laser spectrum was $4.4\times 10^{5}$. This shows that, compared with the interference spectrum, although the RI sensitivity of the laser-based sensor remained unchanged, the resolution did improve by four orders of magnitude. This is exactly the advantage of laser demodulation, which has been proved by some research [48]. Each very small shift would be distinguished. Thus, laser-based sensing methods were suitable for low-concentration biosensing. Thus, the laser spectral demodulation method was adopted in the following protein-detection experiments.

**Figure 4:**
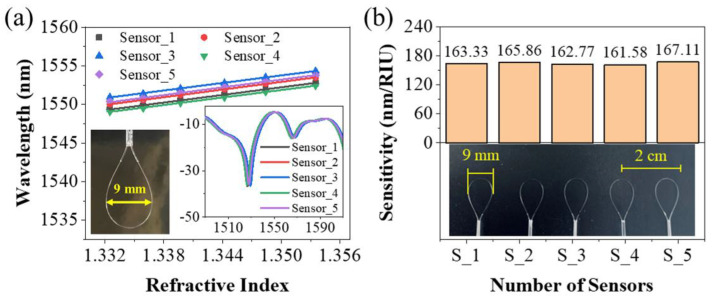
Five lasso structures with bending diameters of 9 mm were made in the same process. (**a**) The laser wavelength of five sensors corresponding to RIs varying from 1.3325 to 1.3535, as well as their linear fitting. Inset: The interference spectra of these five lasso structures. (**b**) The sensitivities of these five sensors fabricated in the same process, which was almost the same.

Other than this, the reproduction of our lasso-shaped fiber sensor was also demonstrated. Five lasso structures with bending diameters of 9 mm were made using the same process. Their interference spectra had the same characteristics, and the RI sensitivities were almost the same, as shown in Figure 4.

### 3.2. Protein Detection Using the Sensing System

**Figure 5:**
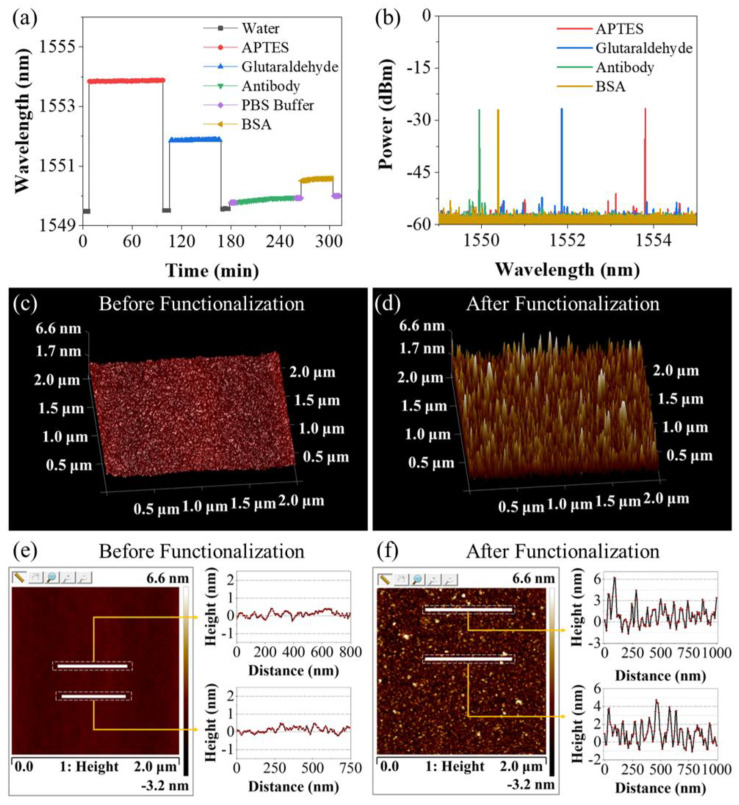
(**a**) Laser wavelength variation of lasso-shaped fiber sensor during the procedures of biofunction. The wavelength changes before and after each stage indicate the alteration of the surface state. (**b**) Laser spectra when the sensor was soaked in four solutions during modification. Atomic force microscopy image of fiber surface (**c**) before and (**d**) after biofunction. Two-dimensional scanning height results of random sampling in the AFM scanning area of fiber surface (**e**) before and (**f**) after biofunction.

The functionalization of the sensor was described in Section 2.2. Figure 5a shows the variation in the laser wavelength of the lasso structure probe during the procedures of functionalization. From the DI water to the APTES ethanol solution, the laser wavelength suddenly jumped up by 4 nm due to the increase in RI. After this step, the lasso structure was washed in DI water again, and the laser wavelength fell back. However, compared with the DI water of the previous stage, the laser wavelength has a redshift phenomenon of about 35 pm, indicating the silanization of the fiber surface. From water surrounding to glutaraldehyde solution, the latter owns a higher RI and causes a wavelength shifting of over 2 nm. After rinsing with the DI water, the actual wavelength shift after soaking was observed to be ~40 pm, denoting the alteration of the surface state. When the fiber probe was soaked in the anti-CEACAM5 antibody solution, continuous red shifting of 0.18 nm could be observed, which demonstrated the success of antibody bonding. During the first 10 min of the BSA blocking process, the laser wavelength shifted by about 70 pm, indicating that the BSA has successfully occupied the vacant aldehyde groups on the fiber surface. After about 15 min, the laser wavelength stabilized, indicating that the BSA blocking process had been completed. Figure 5b presents the laser spectrum when the lasso-shaped fiber probe was immersed in these four solutions.

To validate the functionalization, atomic force microscopy (AFM) was employed. The detailed method and the instrument information are described at the end of Section 2.2. After drying for 12 h, the fiber sensor was imaged with AFM and compared with the optical fiber without biological modification. Figure 5c,d present the surface feature of the bare fiber (the coating layer was removed) without biofunction and after biofunction. It can be found that, compared with the surface state of the fiber before biofunctionalized modification, the fiber after functionalization had a larger non-uniform surface with a higher perturbation ratio. The two-dimensional height image of Figure 5c is shown in Figure 5e, and the height information at the position of two white lines (random sampling) is also given. The results showed that the surface of the fiber before biofunction was flat, and the height fluctuation was less than 0.8 nm. Similarly, Figure 5f presents the two-dimensional height image of the fiber surface after functionalization as well as the height information at the position of two white lines (random sampling). The results showed that the height fluctuation of these positions basically reaches 3~4 nm, and some positions even have a height difference of more than 6 nm. These results strongly suggest that a layer of material with longitudinal dimensions of a few nanometers was attached to the surface of the optical fiber, which also matches the size of the protein molecules.

**Figure 6:**
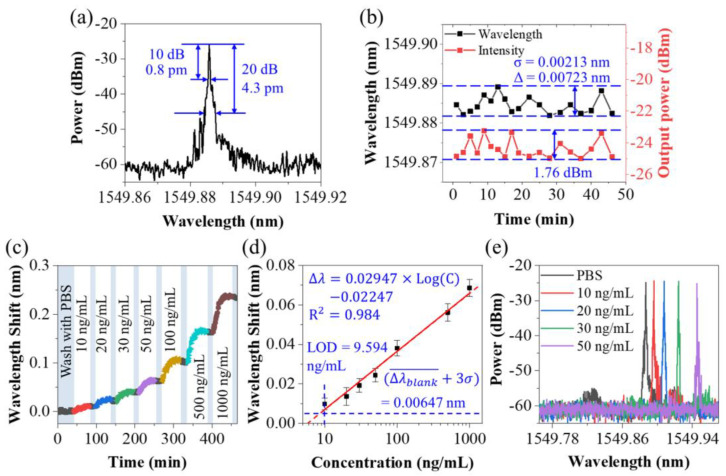
(**a**) Laser spectrum corresponding to the lasso structure measured with BOSA, when the fiber sensor was soaked in the PBS buffer. (**b**) Time series recording of laser wavelength and laser power corresponding to the sensor immersed in the PBS buffer. (**c**) Laser wavelength shift during the detection process of CEACAM5 with various concentrations ranging from 10 ng/mL to 1000 ng/mL. (**d**) Response and calibration curves of the biosensor to different concentrations of CEACAM5 protein molecule. (**e**) Laser spectra of fiber sensor in CEA protein solutions with concentrations varying from 10 ng/mL to 50 ng/mL after being sufficiently combined.

The lyophilized CEACAM5 protein was reconstituted to various concentrations ranging from 10~1000 ng/mL using the PBS buffer. Prior to the detection of CEACAM5 protein solutions, PBS buffer (blank control) was tested long-term to see the wavelength shifts. Figure 6a shows the laser spectrum when the fiber probe was soaked in the PBS buffer. Figure 6b presents the wavelength and power fluctuation of the laser signal in this period. During nearly 50 min of monitoring, the maximum shift of wavelength and the intensity of the laser were 7.23 pm and 1.76 dBm. The mean wavelength variation of the blank control group (PBS buffer) $\overset{-}{∆\lambda_{blank}}$ and the standard deviation *σ* of the random fluctuation were measured to be 0.08 pm and 2.13 pm, respectively.

While detecting CEACAM5 solutions from low concentration to high concentration, after each detection, the sensor was rinsed in PBS buffer for 10 min to remove non-specific or unstable binding. BOSA was used to capture the tiny variation during the immune binding, as shown in Figure 6c. For these each concentration, the laser wavelength had an observable response. The response curve of the laser-based biosensor regarding the alteration of the CEACAM5 concentration is given in Figure 6d. Three independent tests were repeated to get the mean wavelength drift and error bar. A log-linearly calibrated curve could be drawn as

(8) ∆λ=0.02947×log⁡C−0.02247

presenting a linear slope of 0.02947 nm/(ng/mL) ranging from 10 ng/mL to 1000 ng/mL, with a linear fit of 0.984. Here, *C* is the concentration of CEACAM5 protein (ng/mL).

The limit of detection (LOD) can be expressed as:

(9) $$LOD=\frac{{\left\{\overset{-}{∆\lambda_{blank}}+\begin{array}{cc} 3\times \sigma , & Resolution \end{array}\right\}}_{MAX}}{S}$$

According to Equation (9), the LOD of our laser-based biosensor for CEACAM5 in buffer was 9.594 ng/mL. Considering that the molecular weight of CEA protein was about 200 kD, our LOD can reach 48 pM.

Figure 6e shows the laser spectra of our fiber sensor in CEA protein solutions with concentrations varying from 10 ng/mL to 50 ng/mL after being sufficiently combined. Thanks to the narrow linewidth characteristic of the laser spectrum, small shifts in the laser spectrum, even as small as 0.01 nm, were easy to distinguish. This is exactly the advantage of laser demodulation. In some published papers, despite the high RI sensitivity of the sensor, the spectrum used for signal demodulation was very flat, and it was difficult to resolve small spectral shifts, which severely limits the LOD of the sensor [49]. In this work, although the RI sensitivity of our sensor was not high, based on the narrow linewidth and high-resolution characteristics of the laser spectrum, our design still showed good detection ability.

Table 1 shows a comparison of LOD and other various parameters from some reported optical fiber biosensors and from this work. Through comparison, it can be found that the RI sensitivity of the sensor was not the only factor that determines the detection capability or LOD of the biosensor. For example, the sandwich strategy can be used to amplify the response of biomolecular binding [50]. Reducing the spectral linewidth and improving the resolution of the demodulation equipment can also make a sensor with low sensitivity display good biological detection ability [9,33]. Although the RI sensitivity of the hyperbolic metamaterial-based sensor was nearly 30 times higher than ours, the LOD was basically an order of magnitude (10 pM vs. 48 pM). Thanks to the narrow linewidth of the laser spectrum in our work, the ability of our sensor to resolve small spectral shifts was greatly improved. Thus, a fiber laser-based lasso-shaped biosensor with about 160 nm/RIU sensitivity was able to approach the performance of biosensors with more than 1000 nm/RIU present in the literature. Of course, the improvement of sensing sensitivity is necessary. If the sensitivity of our sensors can be further improved, it will certainly be possible to detect lower concentrations of biomolecules, which was one of the subsequent improvement directions.

**Figure 7:**
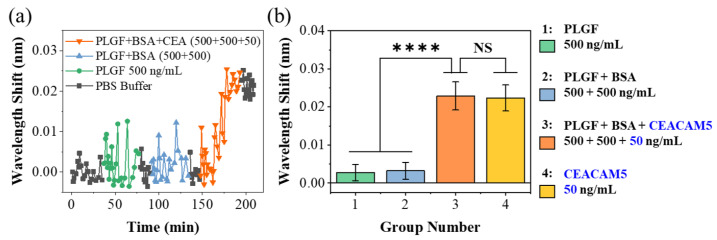
(**a**) The laser wavelength changes during the detection of three control groups. (**b**) Bar graph of laser wavelength shift in these control group tests. The test objects of groups 1–4 are listed on the right. The significance tests of difference between the detection results of groups 1–4 were made. **** indicated the hypothesis testing with *p* < 0.0001; NS indicated the hypothesis testing with *p* > 0.05 (0.6106).

To prove the specificity of the probe, we utilized two kinds of non-specific proteins— PLGF and BSA—and designed three different proportions of protein PBS solution as controls. The first was to configure lyophilized PLGF with PBS buffer to a concentration of 500 ng/mL. In the second group, PLGF and BSA were added to the PBS buffer, and the concentrations of both were 500 ng/mL. In the last group, CEACAM5 protein with a concentration of 50 ng/mL was added to PLGF and BSA mixture solution. The sensing probe was sequentially immersed in three different solutions for 45 min. Before and after each test, the surface of the probe was immersed in PBS buffer for washing the biological residues. Figure 7a shows the variation of laser wavelength during the whole biological testing period. In the previous two controls, the fluctuation range of laser wavelength was significantly larger than that in pure buffer (0~35 min) due to the random movement of biomolecules and moving close to the fiber probe. However, because these biomolecules did not match the anti-CEACAM5 antibody on the surface of the sensor, this increase in laser wavelength (non-specific binding) fell back after washing in PBS buffers (80~90 min and 135~145 min). However, after the third test group, the laser wavelength (195~205 min) increased significantly by about 0.02 nm, which demonstrated the specific binding of CEACAM5 protein in this mixture solution. Three independent tests were repeated to get the mean wavelength drift and error bar.

Figure 7b presents the laser wavelength variation in each control experiment, and the wavelength variation in pure CEACAM5 PBS solution (50 ng/mL) was also given in the figure for comparison. The control groups without CEA protein caused a small spectral response of 2.74 pm and 3.21 pm, respectively. The molecular concentration of PLGF and BSA was 10 times that of CEACAM5 protein; however, once CEACAM5 protein (only 50 ng/mL) was added to the mixture solution, the wavelength variation was significantly increased to 23.1 pm. This wavelength offset was comparable to that of 50 ng/mL CEACAM5 PBS solution. The significance tests of difference between the detection results of different groups were also made. **** indicated the hypothesis testing with *p* < 0.0001, showing a great significance between groups with and without the CEACAM5 molecule. NS indicated the hypothesis testing with *p* > 0.05 (0.6106), showing no significance between pure CEACAM5 solution and protein mixture solution with CEACAM5 protein. The experimental results fully demonstrate the specific detection ability of our sensor.

**Figure 8:**
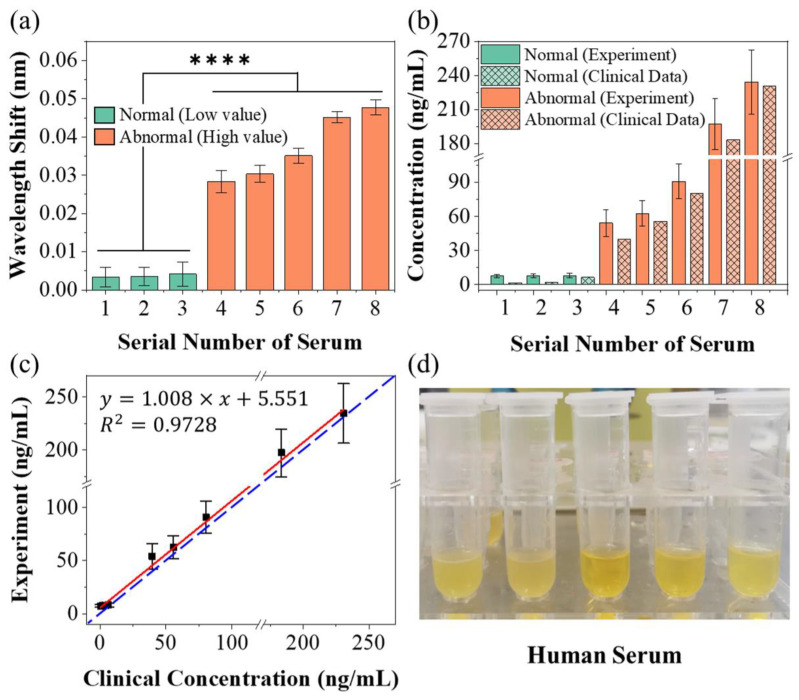
(**a**) Responses of the laser-based lasso-shaped biosensor to different samples of human serum with low (green marker) and high (orange marker) CEACAM5 concentration; **** indicated the hypothesis testing with *p* < 0.0001. (**b**) Comparison chart of CEACAM5 concentration in serum samples calculated (solid color) according to the Equation (9) and clinical data (grid marked). (**c**) Comparison and linear fitting between the clinical data and measured values in this experiment. (**d**) Photograph of human serum samples to be tested.

To further verify the sensing capability of our laser-based biosensor, the detection was carried out on human serum samples obtained from the hospital. Figure 8a presents the wavelength shift of our biosensor to different samples of human serum. According to the clinical data, human serum samples were divided into two groups based on the concentration of CEACAM5. For the serum samples with low concentration (marked with green), the response of the biosensor was very weak, with the wavelength offset less than 0.01 nm. For serum samples with high concentration (orange marked bars), the wavelength offset was dramatically increased. The result of the significance test showed a great significance of the test (statistical hypothesis: *p* < 0.0001, marked as ****) between serum samples with low and high CEACAM5 concentration. The experimental results prove that our sensor is capable of distinguishing low CEA concentration from high CEA concentration in serum samples.

According to Equation (8), the concentration of CEACAM5 can be inversely deduced. The calculated CEACAM5 concentrations (solid color marked bars) of these serum samples were compared with clinical data (grid marked bars), as shown in Figure 8b. The specific numerical results of wavelength offsets and concentrations derived from Equation (8) are given in Table 2 and compared with clinical data. During the testing of the first three groups of serum samples, because the CEA concentration was very low, even lower than the LOD of our sensor, the wavelength offset did not change much and was similar to the offset of the blank control group. The concentration values of these three groups derived from Equation (8) are not strongly related to clinical data, but kept low, which is in sharp contrast with other groups with higher concentrations. For the remaining groups of serum samples, because CEA concentrations were higher than the LOD, the wavelength shift response became obvious. There is a similar trend between our detection results and clinical data, that is, the higher the CEA concentration (clinical value) in serum samples, the higher the results we measured. Our results are slightly higher than the clinical data, which may be due to the more severe non-specific adsorption in the complex serum environment. In addition, since the inversion of Equation (8) from wavelength to concentration was exponential, a similar wavelength standard deviation (SD) causes a larger SD of concentration when the wavelength was larger. Figure 8c shows the linear fitting between the clinical data and measured values in this experiment. The horizontal axis represents the clinical data provided by the hospital, and the vertical axis represents the value measured by the experiment and deduced by Equation (8). Ideally, the experimental measurements are exactly equivalent to the clinical data (the blue dashed line: *y* = *x* in Figure 8c). The truth is that our experimental results are slightly off from the clinical data. The slope of the linear fit (red line) is 1.008 with a fitting bias of 5.551 and a fit degree of 0.9728. The slope of the linear fit is close to 1, indicating that our test results have a similar trend to the clinical data. The slight deviation was due to more severe nonspecific binding in the serum. This deviation can be removed by making a difference in the measurement results of the blank group to achieve a certain degree of compensation. Our biosensor can maintain similar sensing performance in different liquid environments (PBS buffer and serum), which may also reflect the advantage of the structural stability of our lasso-shaped biosensor to some extent. Figure 8d shows the photograph of human serum samples to be tested. All in all, these results fully demonstrated the excellent bio-detection capability of our laser-based lasso-shaped biosensor, even in the complex environment of human serum.

## 4. Conclusions

In this paper, a fiber laser-based lasso-shaped biosensor was proposed for the specific detection of CEACAM5 protein in serum and demonstrated good repeatability as well as high specificity. Due to the ultra-high optical resolution and ultra-narrow linewidth of laser, the advantage of narrow linewidth and high-precision sensing capability of laser can be fully observed. Each small offset caused by biomolecular bonding can be clearly distinguished. Functionalization of the sensor was realized to detect a kind of cancer biomarker—CEACAM5. The limit of detection (LOD) could reach 9.6 ng/mL in buffer. The specificity of the biosensor was verified in control experiments. Human serum samples from a hospital were also tested. Our laser-based biosensor can effectively distinguish between low and high concentrations of CEACAM5 molecules in serum. Several approaches can be anticipated in order to further enhance the sensing capability of our biosensor, such as using localized surface plasmon resonance and tapered fiber to improve the sensitivity and using the antigen sandwich method to amplify the signal [51,52]. Furthermore, fiber Bragg grating can be added to the fiber ring cavity to realize multi-parameter detection and temperature compensation [53]. The photothermal effect of laser would be the next direction of our research [54,55]. This sensor holds great potential to serve as a POCT device, which can be extended to a broader field of biological detection and environmental monitoring.

## Figures and Tables

**Table 1:** Comparison of LOD and other various parameters of some reported optical fiber biosensors and this work.

Method	RI Sensitivity	FWHM	FOM	LOD	Reference
hyperbolic metamaterial and graphene film on fiber	4461 nm/RIU	>200 nm	~20	10 pM	[49]
SPR and AuNPs Amplification (Sandwich model)	1452 nm/RIU	~100 nm	14.5	1 pM	[50]
Microfiber Bragg grating (mFBG)	60 nm/RIU	~2 nm	30	13.5 ng/mL (365 pM)	[9]
mFBG and microwave photonics filter (MPF) demodulation	7 nm/RIU (OSA) 255 GHz/RIU (MPF)	~0.2 nm 26 MHz	35 (OSA) $9.8\times 10^{3}$	50 ng/mL (270 pM)	[33]
Lasso structure and laser demodulation	164 nm/RIU	<0.8 pm	$4.4\times 10^{5}$	9.6 ng/mL (48 pM)	This work

**Table 2:** Comparison of the experimental results of human serum samples with clinical data.

Category	Wavelength Shift (pm)		Concentration (ng/mL)		
	Mean Value ^1^	SD ^2^	Mean Value	SD	Clinical Data
Sample 1	3.38	2.19	7.62	1.35	0.55
Sample 2	3.96	2.54	8.01	1.59	1.91
Sample 3	4.77	3.03	8.58	2.00	6.45
Sample 4	28.36	2.88	54.08	12.02	39.72
Sample 5	30.32	2.22	62.64	11.06	55.64
Sample 6	35.09	1.99	90.72	15.01	80.43
Sample 7	45.11	1.47	197.46	22.48	183.51
Sample 8	47.38	1.94	235.99	30.42	230.77

^1^ Take the average of three measurements; ^2^ SD: Standard deviation.

## Data Availability

Not applicable.
